# Supplementary material for: Layers of the monkey visual cortex are selectively modulated during electrical stimulation
Source: PLoS Biol. 2025 Jul 7;23(7):e3003278. doi: 10.1371/journal.pbio.3003278 (PMC12251224; doi:10.1371/journal.pbio.3003278)
Supplement: S1 Text — Contains Figs A–I and Table A. Fig A. CSD and LFP responses across layers. A) Depth profile of the current source density (CSD) signals averaged across trials. The color yellow represents the current sink, while the color black represents the current source. B) LFPs recorded using multisite probe in monkey 2. Only the contacts involved in cortical layers are illustrated. Red lines represent visual-evoked LFPs in the Flash + AC condition, and blue lines depict LFPs in the Flash condition. C) Comparison of layer-averaged amplitudes of LFP components, P1 and N1, between the Flash (blue) and Flash + AC (red) conditions for both monkeys. Fig B. Effects of AC on layer-specific visual-evoked LFPs in V1. A) Monkey 1; B) Monkey 2. Normalized LFPs were averaged across the trials and contacts within each layer. Thick lines represent averaged LFP in the Flash condition (blue) and Flash + AC condition (red), with shades representing the standard deviation. Fig C. Permutation test results for phase-dependent LFP modulation. The permutation analysis for A) monkey 1 and B) monkey 2 corresponds to Fig 3. The gray histogram represents 5,000 permuted vector lengths. Gray dotted lines indicate the significance level. The blue and red lines indicate the unimodal vector length and bimodal vector length obtained from the original data, respectively. For monkey 1, the permutation test reveals significant bimodal phase preference in P1 and N1 with respect to the phase of AC within deeper layers (layers 4–6). For monkey 2, there is a unimodal phase preference in the amplitude of LFP components depending on the phase of AC in the deeper layers. Fig D. Phase-dependent modulation of LFP components under virtual AC. Amplitude of P1 and N1 components according to the phase of virtual AC in the Flash condition for both monkey 1 (A and B) and monkey 2 (C and D). A, C) The P1 and N1 components were sorted into 20 phase bins, followed by taking trial- and phase-averages for each layer. Gray thick and [file pbio.3003278.s003.docx]

**Supplementary Information**

**Layers of the monkey visual cortex are selectively modulated during electrical stimulation**

Sangjun Lee^1*^, Zhihe Zhao^1^, Ivan Alekseichuk^1,2^, Jimin Park^1^, Sina Shirinpour^1^, Gary Linn^3,5^, Charles E. Schroeder^3,4^, Arnaud Y. Falchier^3,5^, and Alexander Opitz^1*^

^1^ Department of Biomedical Engineering, University of Minnesota, Minneapolis, Minnesota, USA

^2^ Department of Psychiatry and Behavioral Sciences, Northwestern University, Evanston, Illinois, USA

^3^ Translational Neuroscience Lab Division, Center for Biomedical Imaging and Neuromodulation, The Nathan S. Kline Institute for Psychiatric Research, Orangeburg, New York, USA

^4^ Departments of Neurological Surgery and Psychiatry, Columbia University College of Physicians and Surgeons, New York, USA

^5^ Department of Psychiatry NYU Grossman School of Medicine, New York City, New York, USA

**^*^** [**lee03936@umn.edu**](mailto:lee03936@umn.edu) **(SL);** **[aopitz@umn.edu](mailto:aopitz@umn.edu) (AO)**


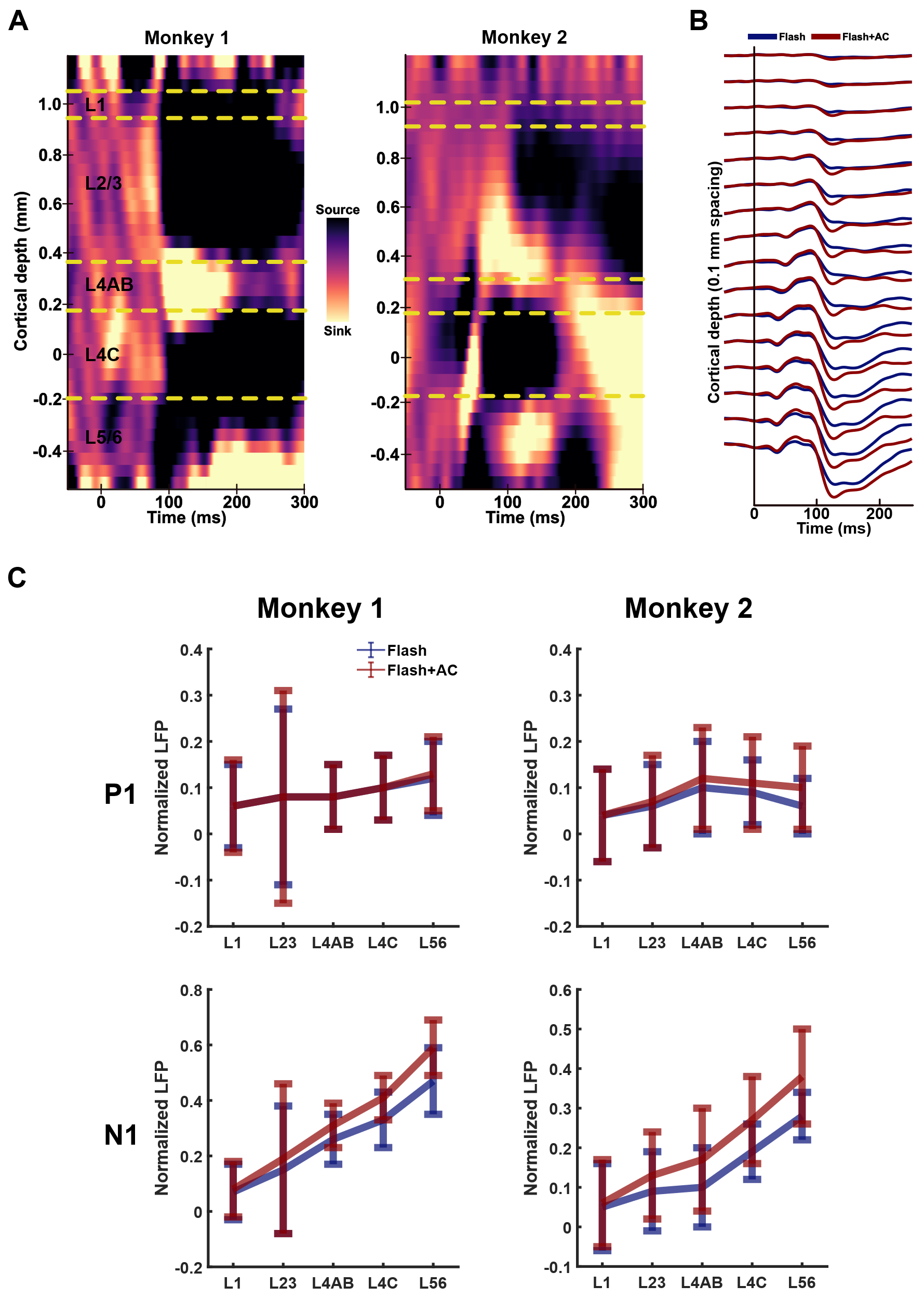


**Fig A. CSD and LFP responses across layers.** A) Depth profile of the current source density (CSD) signals averaged across trials. The color yellow represents the current sink, while the color black represents the current source. B) LFPs recorded using multisite probe in monkey 2. Only the contacts involved in cortical layers are illustrated. Red lines represent visual-evoked LFPs in the Flash + AC condition, and blue lines depict LFPs in the Flash condition. C) Comparison of layer-averaged amplitudes of LFP components, P1 and N1, between the Flash (blue) and Flash + AC (red) conditions for both monkeys.


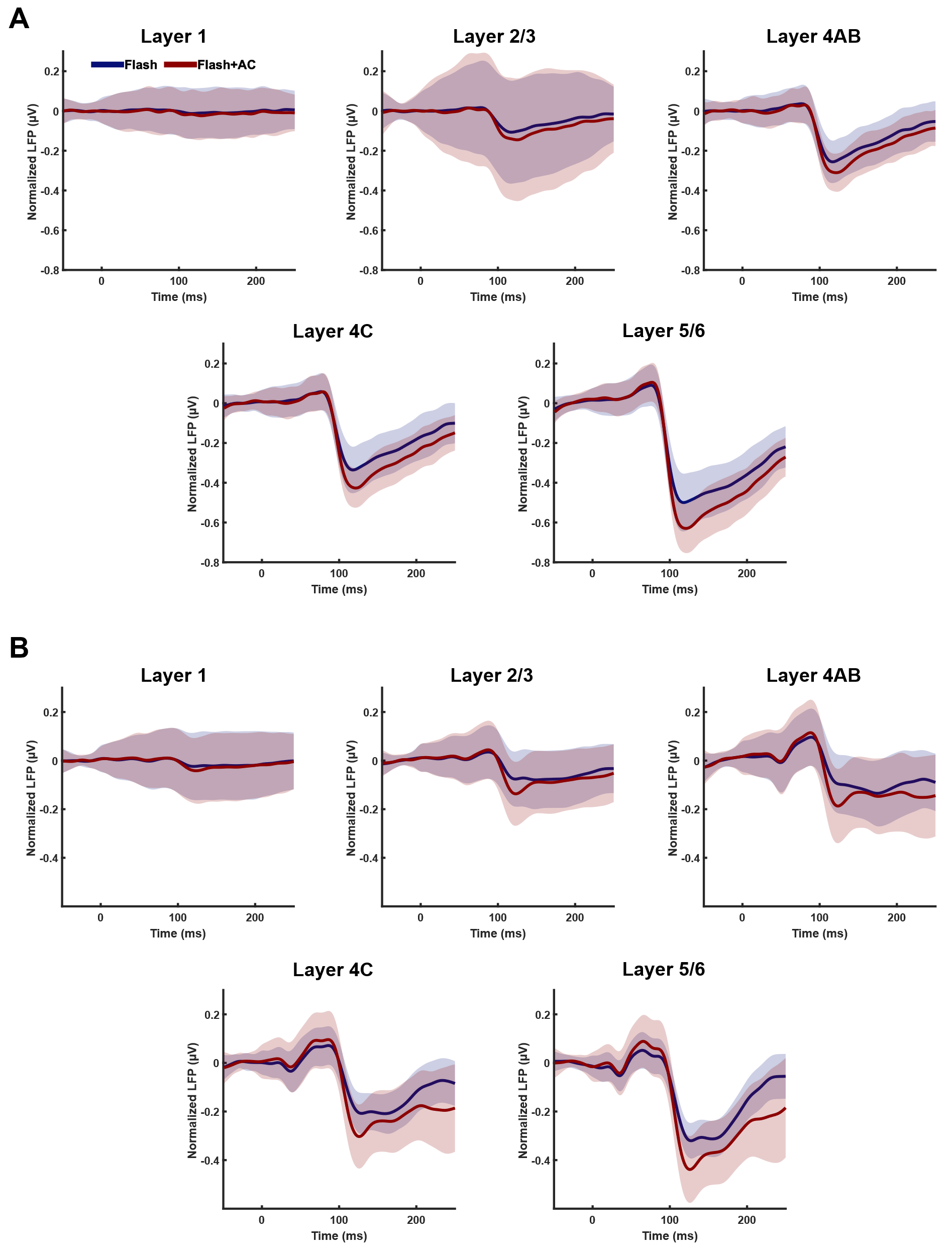


**Fig B. Effects of AC on layer-specific visual-evoked LFPs in V1.** A) Monkey 1; B) Monkey 2. Normalized LFPs were averaged across the trials and contacts within each layer. Thick lines represent averaged LFP in the Flash condition (blue) and Flash + AC condition (red), with shades representing the standard deviation.


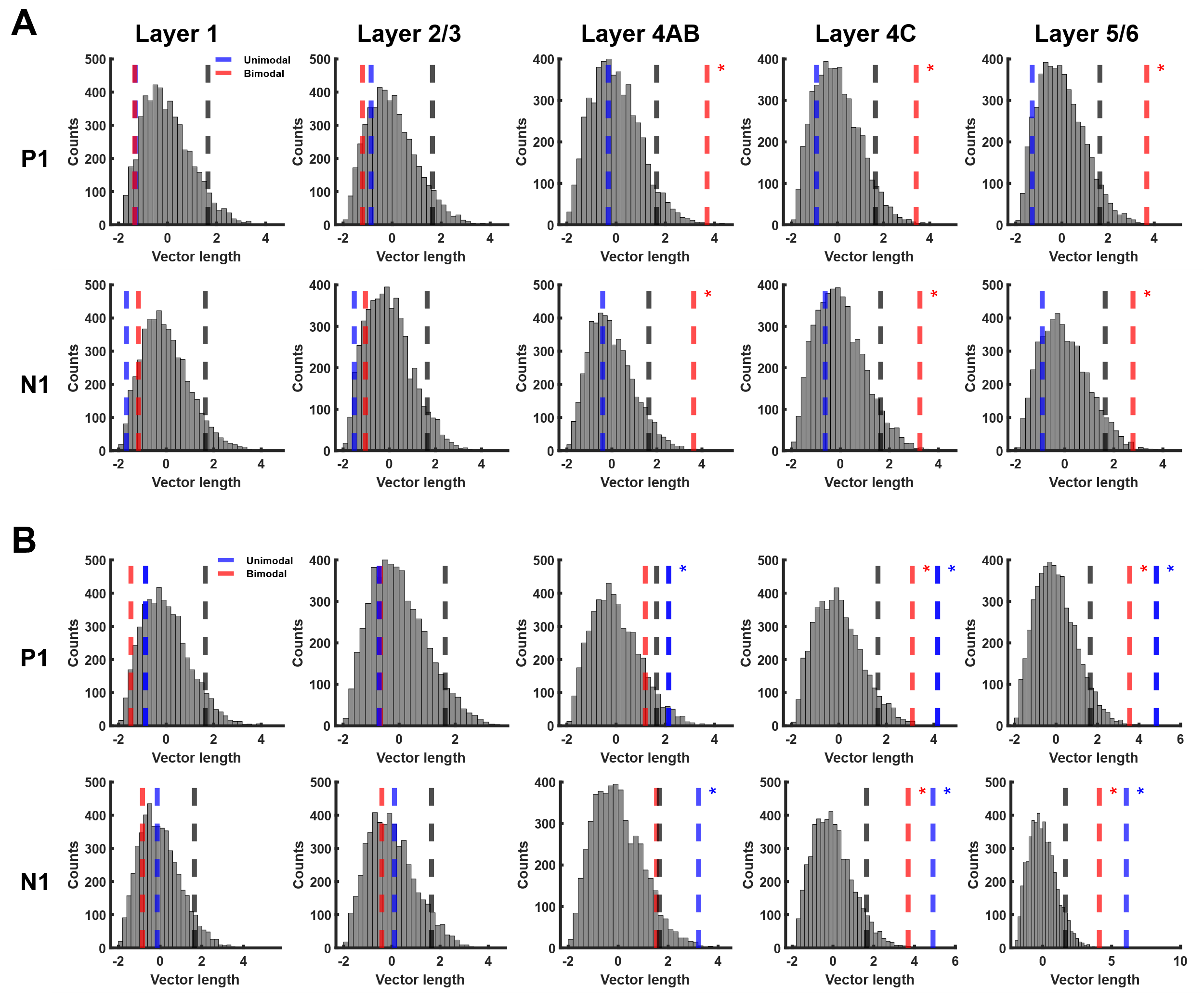


**Fig C. Permutation test results for phase-dependent LFP modulation.** The permutation analysis for A) monkey 1 and B) monkey 2 corresponds to Fig 3. The gray histogram represents 5000 permuted vector lengths. Gray dotted lines indicate the significance level. The blue and red lines indicate the unimodal vector length and bimodal vector length obtained from the original data, respectively. For monkey 1, the permutation test reveals significant bimodal phase preference in P1 and N1 with respect to the phase of AC within deeper layers (layers 4–6). For monkey 2, there is a unimodal phase preference in the amplitude of LFP components depending on the phase of AC in the deeper layers.

**
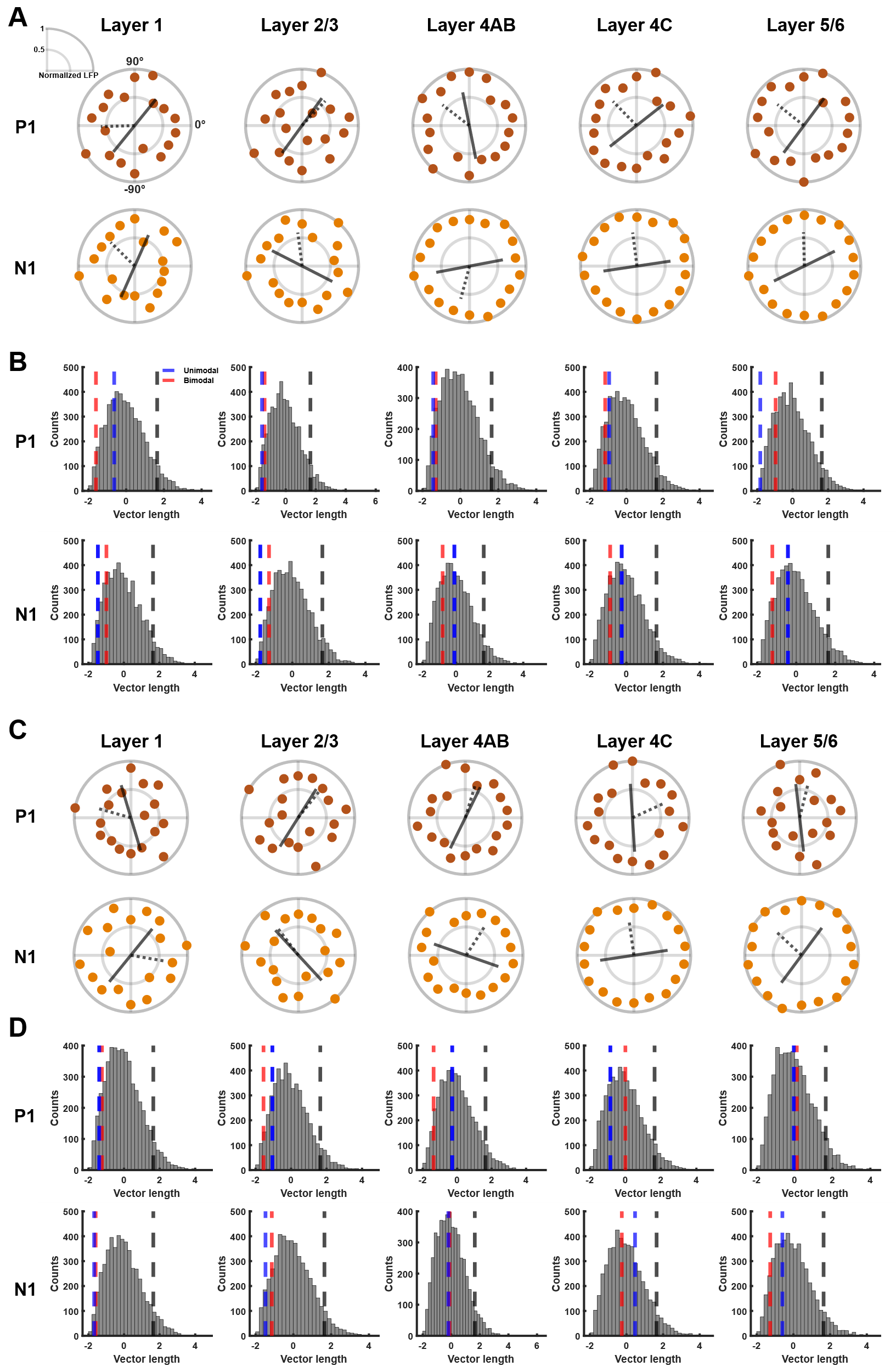
**

**Fig D. Phase-dependent modulation of LFP components under virtual AC.** Amplitude of P1 and N1 components according to the phase of virtual AC in the Flash condition for both monkey 1 (A and B) and monkey 2 (C and D). A, C) The P1 and N1 components were sorted into 20 phase bins, followed by taking trial- and phase-averages for each layer. Gray thick and dotted lines represent the bimodal mean direction and unimodal mean direction of the amplitude of LFP components, respectively, based on the phase of virtual AC. B, D) Permutation test shows the absence of significant directional preferences in the LFP component with respect to the phase of AC across all cortical layers when virtual AC is assumed to be applied in the Flash condition.


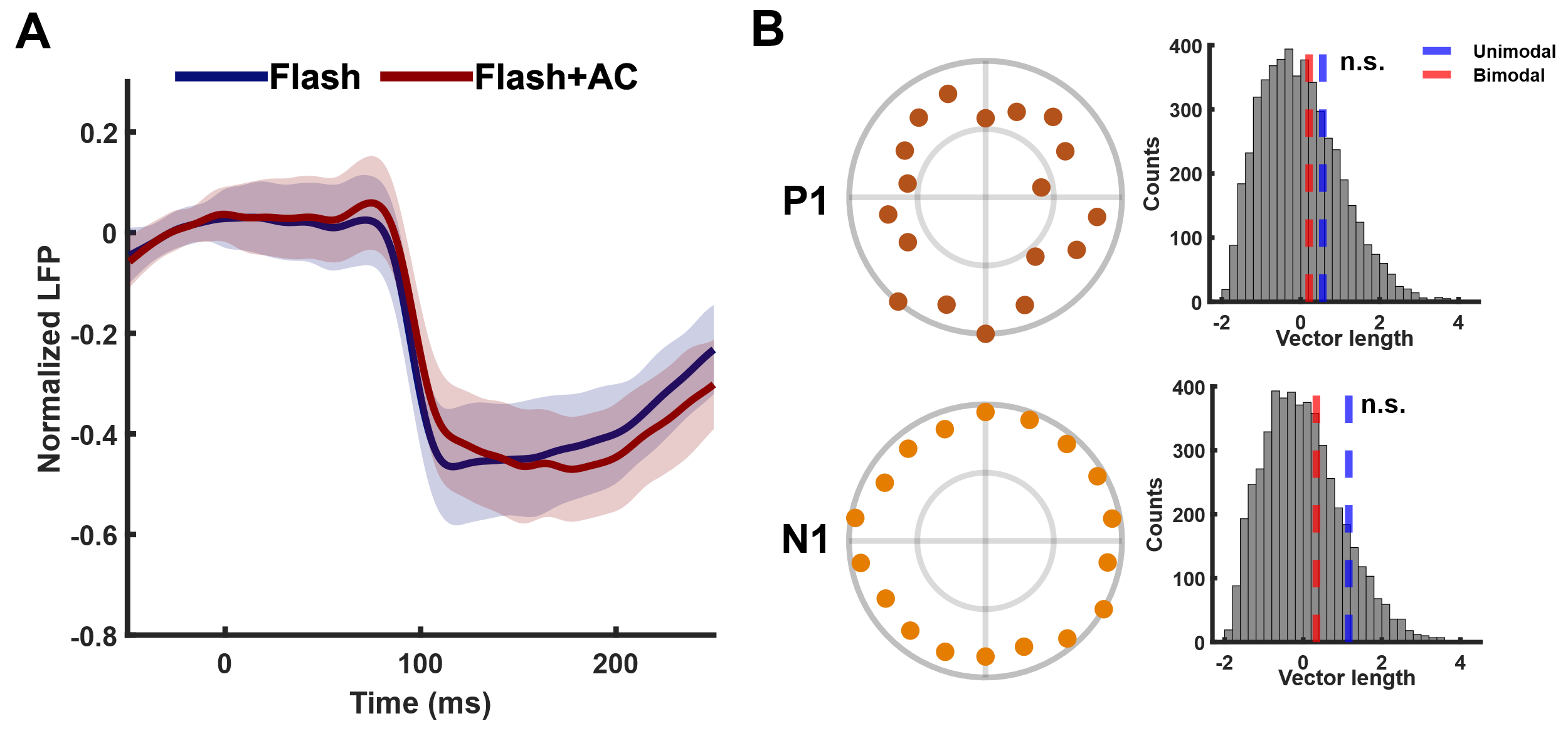


**Fig E. Effect of electrical stimulation on LFP activity in white matter.** A) Local field potentials (LFPs) in white matter in monkey 1. Normalized LFPs were averaged across trials and contacts within white matter. Thick lines and shades represent the averaged LFP and standard deviation, respectively. Time indicates the duration from the flash visual stimulus onset. B) Circular distributions of the amplitude of LFP components, P1 and N1, depending on the phase of AC in white matter in monkey 1 (left column). The results from the permutation test are depicted for P1 and N1 components (right column). The permutation test shows that there is no significant directional preference in P1 and N1 amplitudes with respect to the phase of AC, regardless of whether the circular distribution was unimodal or bimodal (n.s., not significant). The gray histogram represents 5000 permuted vector lengths. The blue lines and red lines indicate the unimodal vector length calculated from the original data and the bimodal vector length, respectively.


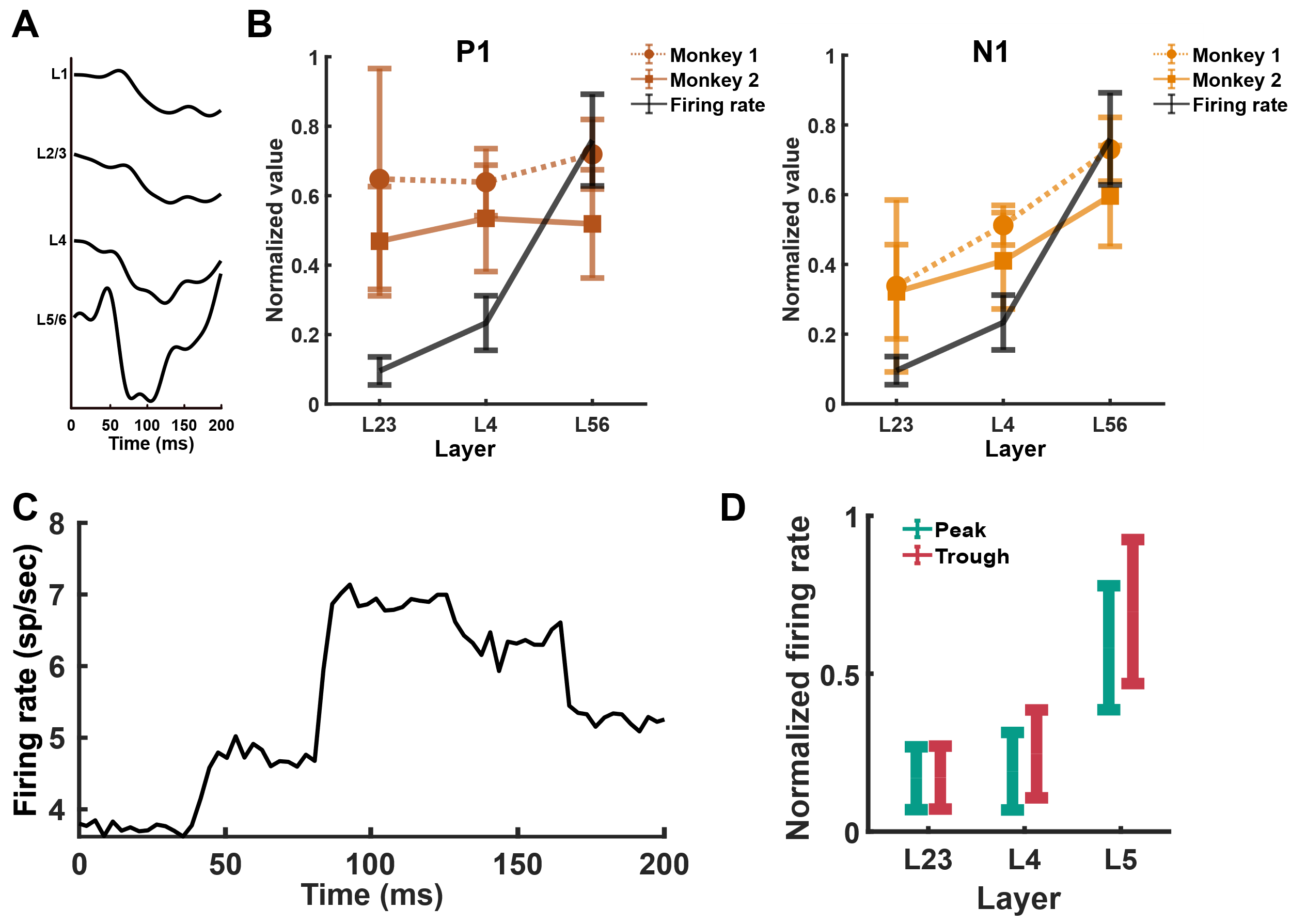


**Fig F. Cortical column modeling results on layer-specific LFP and firing dynamics.** A) LFPs evoked by the flash stimulus in the cortical column model across layers. B) Comparison of LFP components (P1 and N1) obtained in vivo experiments and the firing rate between 50 ms and 150 ms. The P1 component does not correlate with an increase in the firing rate (left), while the N1 component shows a pattern that corresponds more strongly with the increasing firing rate as depth increases in both monkeys (right). To directly compare with the phase dependency of firing rates, only LFP components occurring within ±25° of each tACS phase (rising, falling, peak, trough) were included in the analysis. C) Firing rate of LGN neurons, showing an initial spike around 50 ms (corresponding to the P1 period in simulations), followed by a second firing arising around the period of neural firing of V1 neurons. D) Normalized firing rate of V1 neurons when the visual stimulus was applied at the either peak or trough phase of AC. The firing rate is higher during the trough phase of AC than the peak phase in the deeper layers, while there is no comparable difference in firing rate between two phase conditions in the superficial layer. Underlying data for this figure are provided in S3 data.


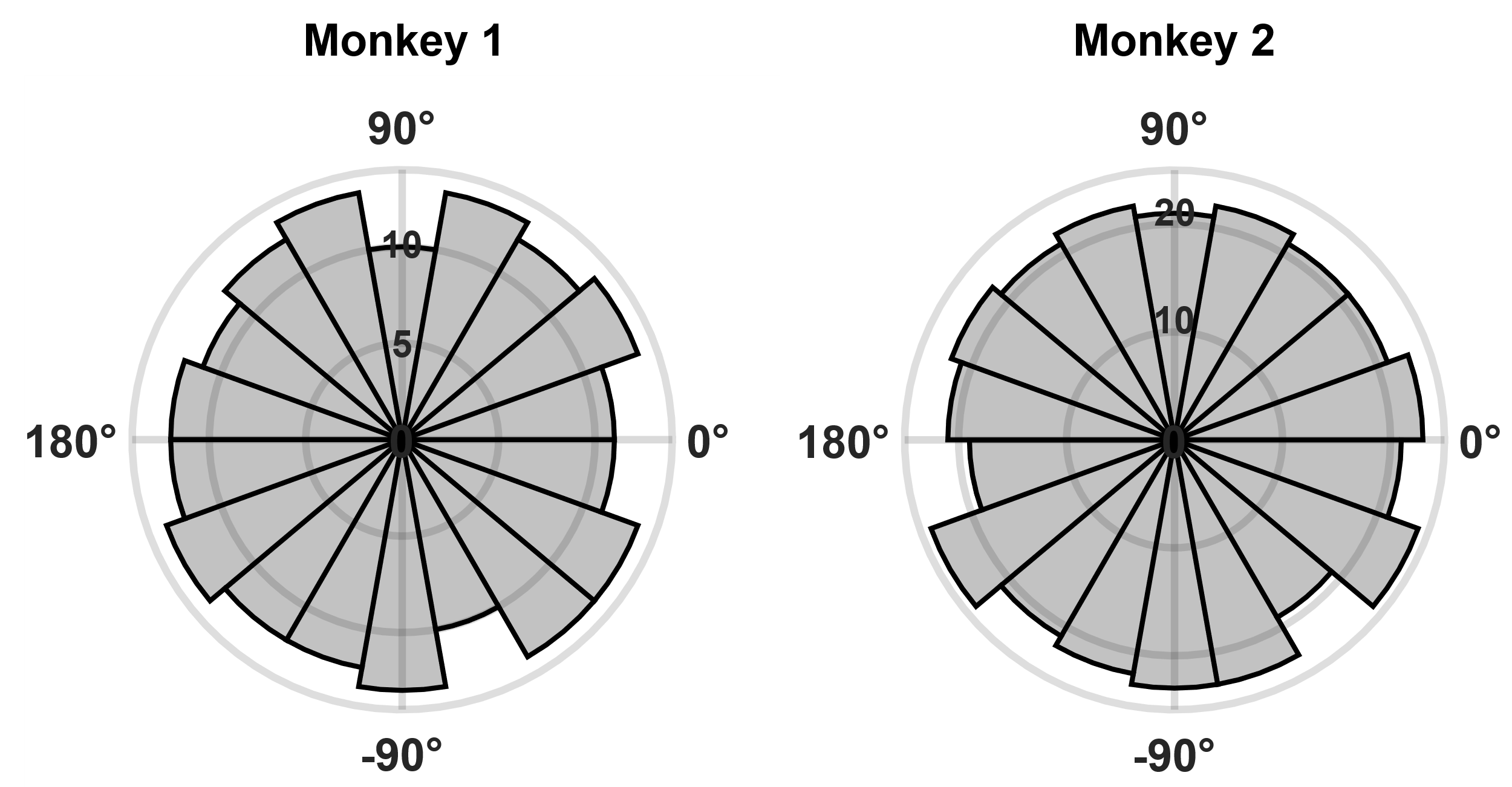


**Fig G. Uniformity of trial phase distribution under Flash + AC condition.** Polar histogram showing the number of trials across phase bins for the Flash + AC condition in both capuchin monkeys. The Rayleigh test confirmed a uniform phase distribution (*p* > 0.05). The average number of trials per phase bin was 12 for Monkey 1 and 21.67 for Monkey 2.


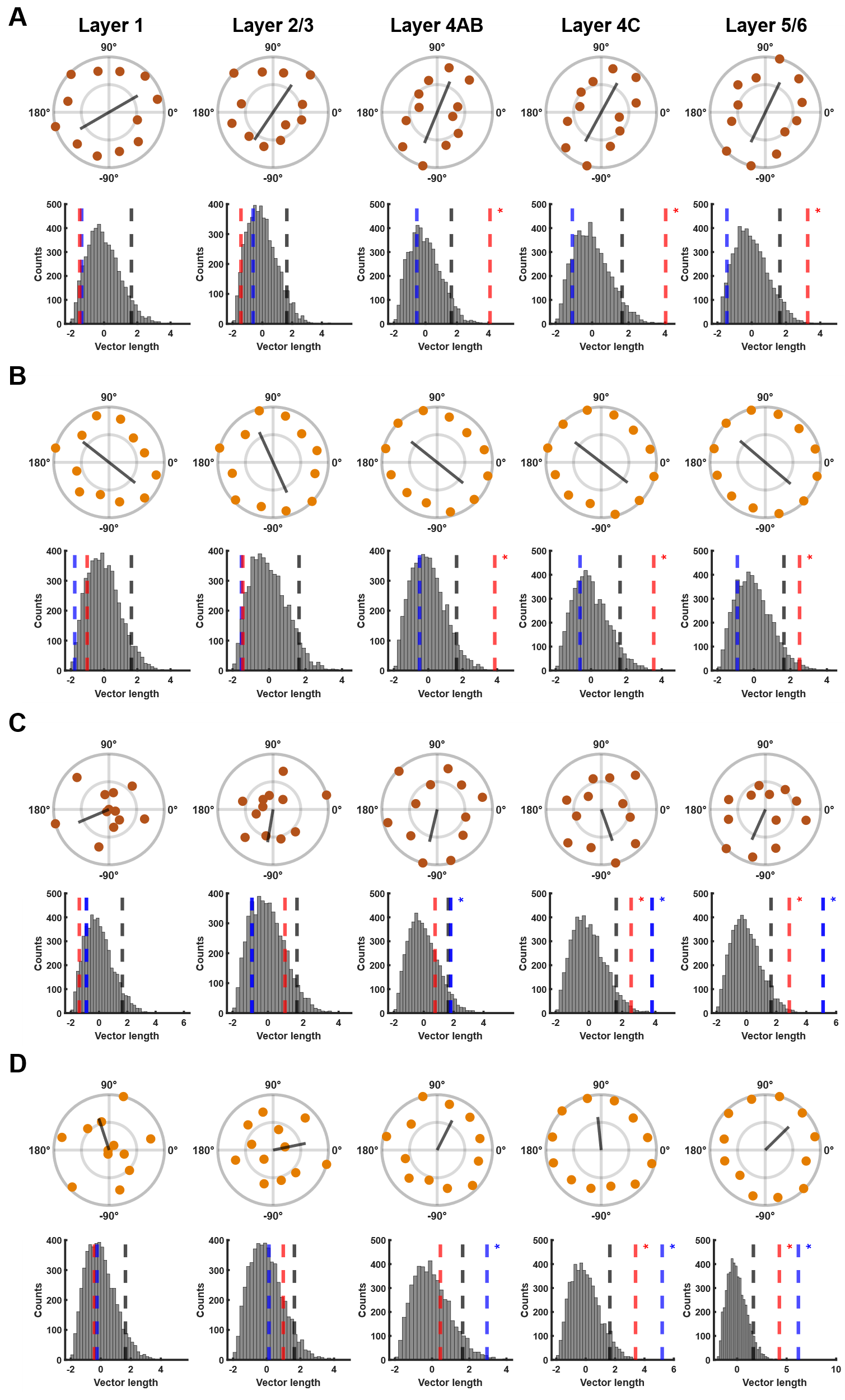


**Fig H. Phase dependency of LFP components with wider phase bins.** Phase dependency analysis using a larger phase bin (30°). Amplitudes of the P1 and N1 components are shown according to AC phases, along with permutation test results. (A, B) P1 and N1 components for Monkey 1. (C, D) P1 and N1 components for Monkey 2.


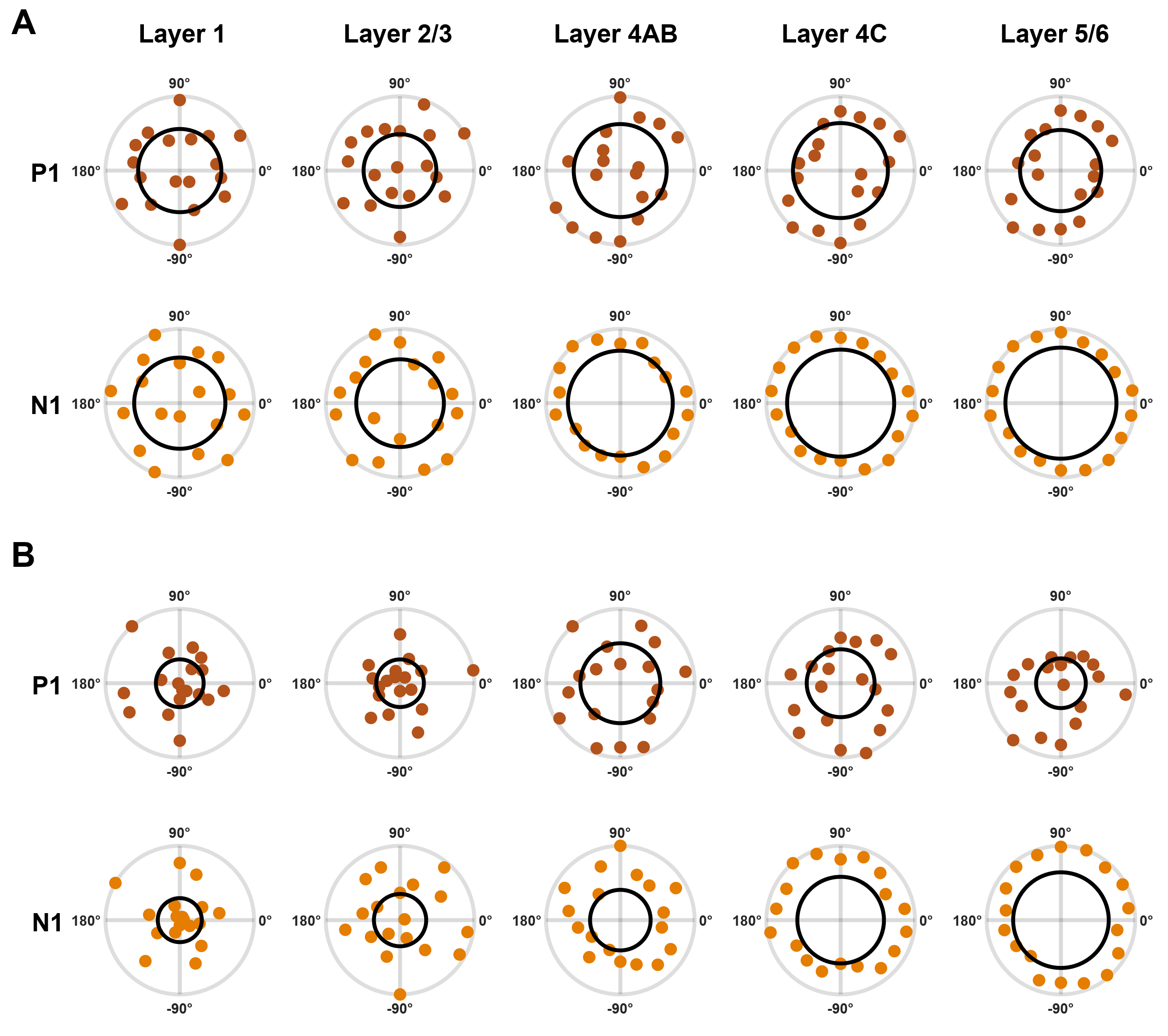


**Fig I. Modulation of P1 and N1 by AC phase relative to Flash baseline.** Phase-dependent modulation of P1 and N1 components under AC stimulation, compared to the Flash condition, in (A) monkey 1 and (B) monkey 2. Black lines indicate the average amplitude of the LFP components in the Flash condition.

**Supplementary Table**

**Table A.** Mean voltage and electric field for each layer in both monkeys.

|  | Monkey 1 | | Monkey 2 | |
| --- | --- | --- | --- | --- |
|  | Voltage (mV) | Electric field (V/m) | Voltage (mV) | Electric field (V/m) |
| Layer 1 | 0.35 | 0.62 | 0.17 | 0.97 |
| Layers 2/3 | 1.05 | 2.53 | 0.70 | 2.76 |
| Layer 4AB | 1.88 | 1.18 | 1.19 | 1.60 |
| Layer 4C | 2.12 | 0.62 | 1.44 | 1.59 |
| Layers 5/6 | 2.32 | 0.37 | 1.76 | 1.42 |
